# Angle-dependent broadband asymmetric acoustic transmission in a planar device

**DOI:** 10.1038/s41598-022-21983-x

**Published:** 2022-11-01

**Authors:** Yuhang Qian, Jing Yang, Jie Hu

**Affiliations:** 1 College of Information Science and Technology, Nanjing Forestry University, Nanjing, China; 2 Key Laboratory of Modern Acoustics (MOE), Institute of Acoustics, Nanjing University, Nanjing, China

**Keywords:** Acoustics, Mechanical properties

## Abstract

Asymmetric manipulation of acoustic transmission is of fundamental interest for wave physics, and has attracted rapidly-growing attentions owing to the potential applications in diverse scenarios. Here we propose to realize angle-dependent asymmetric acoustic transmission by designing a planar structure comprising a gradient-index layer and a layer of homogeneous medium with relatively-lower index. We analytically derive the working frequency and angle range of the device with unidirectional mechanism. And the simulated results show that the proposed device gives rise to high-efficiency broadband asymmetric transmission by allowing acoustic waves normally incident on one side to pass, while behaving as an acoustic barrier blocking waves obliquely incoming from both directions as angle of incidence exceeds a critical angle. Bearing the advantages of simple design, broad bandwidth and switchable functionality, our scheme opens a route to the design of novel acoustic devices capable of adapting various circumstances, and may find applications in noise control, medical detection, etc.

## Introduction

As acoustic counterpart of electric diodes, acoustic one-way devices characterized by unidirectional continuity and reverse cutoff have attracted rapid-growing tensions in the past decades. The realization of such asymmetric transmission for sound is of fundamental interest and may have important implication in diverse applications such as by enabling one-way sound barrier in noise mitigation or by reducing the side-effects of backscattering waves in ultrasound diagnosis and therapy.

Due to the reciprocity principle of acoustic system, it’s difficult to realize asymmetric transmission in traditional acoustic devices. The development of acoustic artificial structures provides a variety of effective methods for designing asymmetric acoustic transmission devices^1–5^. In 2009, Liang et al. combined the nonlinear acoustic materials and linear phononic crystals to realize the one-way flow of acoustic waves for the first time in theory^6^. The corresponding experimental investigation was demonstrated in 2010^7^. Later they develop different mechanisms for producing asymmetric acoustic transmission, like deviated band gap effect of the phononic crystal, acoustic gradient materials, zero-refractive index materials and so on^8–13^. In 2014, Popa et al. presented the idea of using active devices to realize acoustic unidirectional transmission, which introduced nonlinear frequency multiplier circuits and asymmetric acoustic structures to enhance the efficiency of acoustic nonreciprocal transmission^14^. In 2015, Zhu et al. designed a unidirectional acoustic waveguide composed of two metasurfaces with different phase gradients^15^. In 2016, Jiang et al. proposed an acoustic unidirectional system based on an acoustic phase array and metamaterial with the refractive index approaching to 0^16^. Hu et al. utilized the asymmetric polygon-shape and the difference between two gases to realize a one-way acoustic device^17^. In 2017, Li et al. utilized a lossy metasurface to create the asymmetric transmission of acoustic waves in which the incident acoustic waves are no longer limited to vertical incidence, but a large range of incident angles^18^. In 2018, a three-layer labyrinth structure was used to achieve asymmetric transmission by Ju et al.^19^. However, the variation of propagation direction and its influence on the functionality of device, which are of practical significance for the applications of one-way devices, still remains to be explored.

In this paper, we demonstrate an asymmetric acoustic system with a maze structure capable of engineering surface phase gradient, which adds a specific phase delay to the incident waves^20,21^ . When the acoustic waves modulated by the phase gradient penetrate into the structure, the waves along one direction are blocked and those from the other direction can pass through the structure smoothly which makes the asymmetric transmission possible. The simulation results show that the asymmetric device performs well in the range of 18275 Hz to 24882 Hz when the deflection of incident acoustic waves is less than 7°. And the deflection angle above 7° leading to a broadband acoustic barrier from both sides.

## Structure design

**Figure 1:**
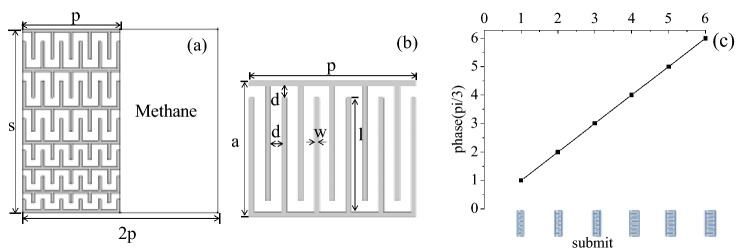
(**a**) Structure of the proposed acoustic device. (**b**) Basic unit of phase gradient structure. (**c**) The phase delay corresponding to each unit.

The design of the asymmetric transmission acoustic device in this paper is shown schematically in Fig. 1a which consists of two parts: 1. a planar phase gradient structure constructed with six basic units which forces the sound to travel in a zigzag way and thus effectively elongates the propagation distance of acoustic waves to provide discrete phase shifts covering the full 2π span with steps of π/3; 2. a layer of homogeneous medium which has the same thickness as the phase gradient by introducing a gas with lower refractive index than air (methane is selected here). Under the guidance of the generalized Snell’s law, a series of coiling labyrinthine metamaterials are deliberately designed to meet the required specifications of phase gradient structure with the length *s* = 1.877 cm and thickness *p* = 1 cm respectively. The parameters of each unit illustrated in Fig. 1b are *p* = 1 cm, *d* = 0.067*p, w* = *0.03p, l* = *a-d-2w,* where *a* is the separation between two neighboring units varying from 0.2*p* to 0.5*p*. The phase delay corresponding to each unit is depicted in Fig. 1c, so that the difference of their modulated phase change can cover a complete 2π range with a single layer and the variation of phase change between adjacent unit cells is about π/3. Then the phase gradient $d\varphi \left(x\right)/dx$ is 2π/s.

## Working mechanism

**Figure 2:**
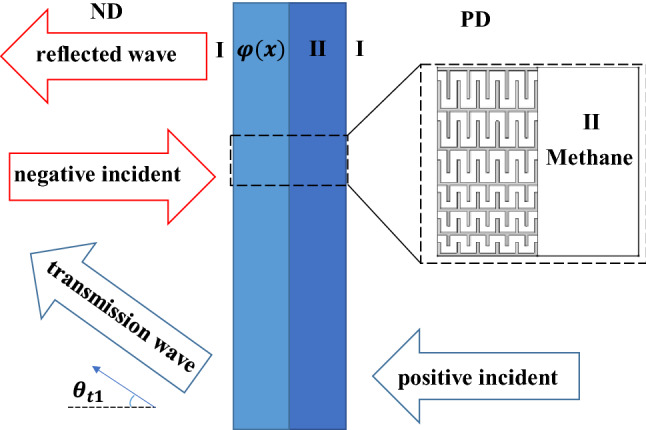
Schematic diagram of the asymmetric transmission structure. The blue arrow and the red arrow are the trajectories of the incident waves from PD and ND respectively.

As shown in Fig. 2, the whole labyrinthine structure is immersed in background air (medium I), and the medium in area II is chosen as methane. $\varphi \left(x\right)$ represents the phase added to the incident acoustic waves by the gradient structure. Here we define the positive direction (PD) as acoustic waves incident from the right side, while the negative direction (ND) is from the opposite side. In order to make the structure achieve asymmetric transmission, we are bound to make sure that acoustic waves can pass through the structure when it is incident along PD, and be blocked along ND.

When the plane acoustic waves penetrate into the structure perpendicularly along PD as shown in Fig. 2, the transmission trajectory will not change at the interface between medium I and II due to perpendicular incidence. However, at the leftmost boundary, the acoustic waves get into air obliquely with the transmitted angle ${\theta }_{t1}$ which is calculated by Eq. (1) shown below:

1 $${ n}_{2}\mathrm{sin}{\theta }_{i}+\frac{\mathrm{d}\varphi \left(x\right)}{\mathrm{d}x}\frac{{\lambda }_{1}}{2\pi }={n}_{1}\mathrm{sin}{\theta }_{t1}$$

where ${\theta }_{i}$ and ${\theta }_{t1}$ are the incident and transmitted angle respectively, the subscript 1,2 refer to different medium in output and incident field. Here ${n}_{1}$=1, ${n}_{2}$=0.73 are the refractive indices of acoustic waves in air and methane, and ${\lambda }_{1}={c}_{\mathrm{A}}/f$ is the wavelength of acoustic waves in which ${c}_{\mathrm{A}}$ is the sound velocity in air,$\frac{\mathrm{d}\varphi \left(x\right)}{\mathrm{d}x}$ is the phase gradient with the value 2π/s, then ${\theta }_{t1}=\mathrm{arcsin}\left[\frac{{c}_{\mathrm{A}}}{2\pi f}\frac{\mathrm{d}\varphi \left(x\right)}{\mathrm{d}x}\right]$ for ${\theta }_{i}=0$. As long as $\frac{\mathrm{d}\varphi \left(x\right)}{\mathrm{d}x}<\frac{2\pi f}{{c}_{\mathrm{A}}}$ is satisfied, the positive incident waves can be transmitted smoothly.

While in the ND case, if the condition $\frac{\mathrm{d}\varphi \left(x\right)}{\mathrm{d}x}>\frac{2\pi f}{{c}_{\mathrm{M}}}$ is satisfied with ${c}_{\mathrm{M}}$ being the sound velocity in methane, there will be virtually no transmitted waves since most of incident energy will be reflected back according to Eq. (1) in which the subscript 1, 2 mean methane and air respectively.

It is obvious that in order to achieve one-way acoustic transmission, the prerequisite to be met is: $\frac{2\pi f}{{c}_{\mathrm{M}}}<\frac{\mathrm{d}\varphi \left(x\right)}{\mathrm{d}x}<\frac{2\pi f}{{c}_{\mathrm{A}}}$ . Given a fixed phase gradient, we can predict the working frequency range as $\frac{\mathrm{d}\varphi \left(x\right)}{\mathrm{d}x}\frac{{c}_{\mathrm{M}}}{2\pi }<f<\frac{\mathrm{d}\varphi \left(x\right)}{\mathrm{d}x}\frac{{c}_{\mathrm{A}}}{2\pi }$. According to the parameters in Fig. 1, the working mechanism will take effect when the frequency ranges from 18275 to 24882 Hz. By rationally designing the phase gradient structure and choosing the media on both sides, the acoustic waves from PD is allowed to pass through the structure while the waves from ND will be blocked under this condition.

## Results and discussion

To verify our proposed mechanism discussed above, we have carried out a series of numerical simulations by using the finite element method, and typical results will be described as follows.

**Figure 3:**
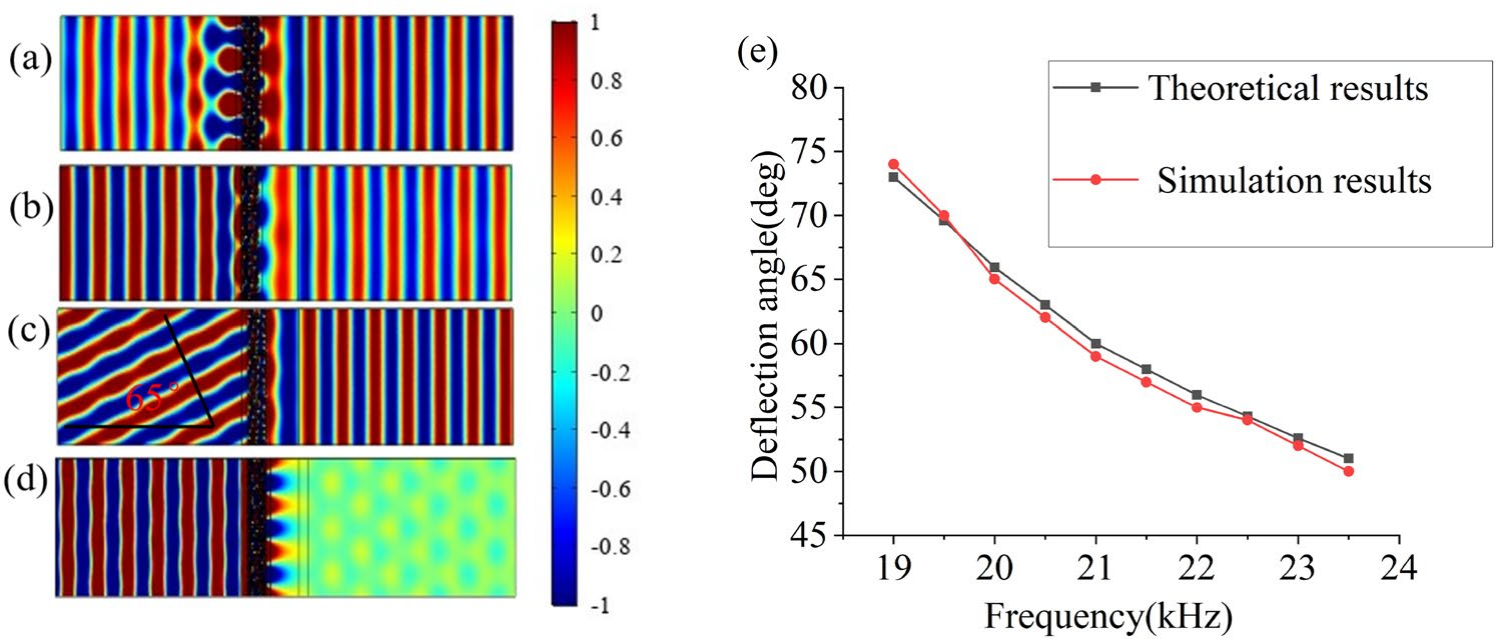
Spatial distribution of the acoustic pressure generated by the incident plane waves propagating along PD (**a**) and ND (**b**) at 17500 Hz, PD (**c**) and ND (**d**) at 20000 Hz. (**e**) Comparison of theoretical and simulated results from 19 to 23.5 kHz.

Figure 3a,b depict the acoustic fields of PD and ND with the acoustic waves impinging normally at 17.5 kHz. Apparently, acoustic waves can pass through from both PD and ND, which means that there’s no asymmetric transmission outside the effective operating frequency band. The acoustic fields of PD and ND at 20 kHz are plotted in Fig. 3c,d. Figure 3c shows that acoustic waves incident along PD effectively passes through air, methane and gradient structure successively and then reaches the output region with the refraction angel ${\theta }_{t1}=$ 65°. In contrast, the acoustic waves incident from the left side along ND are blocked (as shown in Fig. 3d) which is consistent with the theoretical predictions. The validity of our mechanisms is also demonstrated by the good agreement in Fig. 3e that illustrates the comparison between the theoretical and numerical results of deflected angles within the frequency range from 19 to 23.5 kHz.

Due to the subwavelength size of the metamaterial unit cell and large transverse size of the whole structure in terms of wavelength, it is sufficiently accurate to treat the acoustic field in our model as plane waves. Hence here we define ${T}_{PD}$ and ${T}_{ND}$ as the energy transmittance for PD and ND respectively. To better understand the effectiveness of asymmetric transmission of the structure in the range of valid, the acoustic transmission contrast ratio ${r}_{c}$ based on ${T}_{PD}$ and ${T}_{ND}$ is introduced to measure the acoustic one-way performance:

2 $${r}_{c}=\frac{{T}_{PD}-{T}_{ND}}{{T}_{PD}+{T}_{ND}}$$

Obviously, the closer the contrast ratio ${r}_{c}$ is to 1, the better the asymmetric transmission performance is.

**Figure 4:**
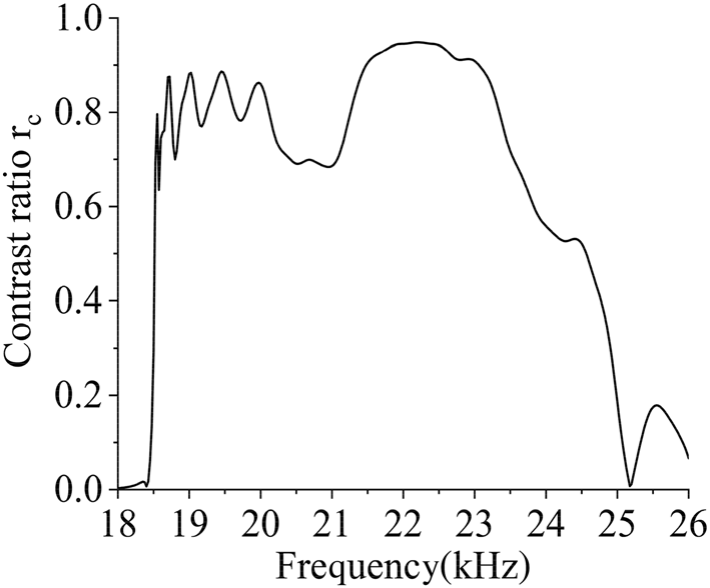
Contrast ratio varies with frequency.

Figure 4 clearly show the asymmetric transmission of acoustic waves within a broad frequency range of 18.5–23.5 kHz where a high contrast ratio ${r}_{c}$ persists which nearly reaches 0.8. The simulated working band is slightly narrower than the theoretical prediction, which is primarily due to the high-order modes of acoustic waves in high frequency.

**Figure 5:**
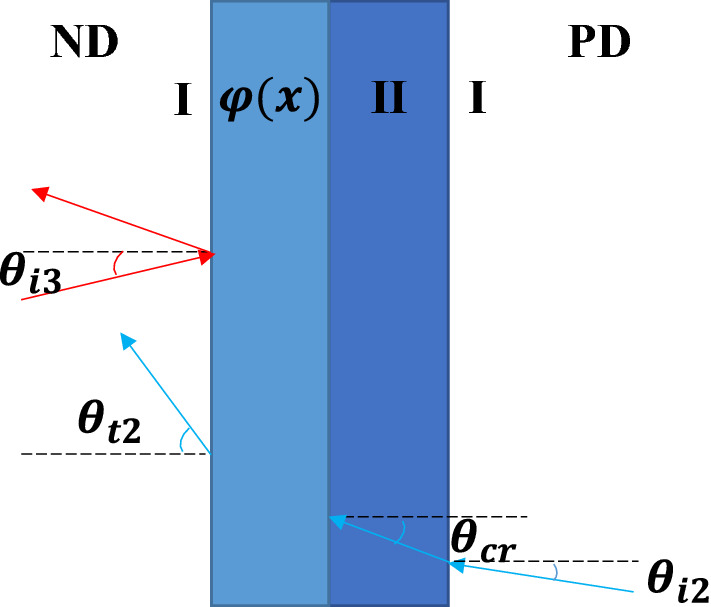
Schematic diagram of asymmetric transmission structure with oblique incidence. The blue path and the red path are the trajectories of the incident waves from PD and ND respectively.

The existing studies of acoustic asymmetric transmission usually focuses on the cases of normal incidence, whereas in practical application the direction of incident waves impinging on such devices may vary largely. To solve this problem, the acoustic waves from different incident angles should be taken into account. Here we take acoustic waves with a center frequency of 20 kHz as an example. As marked by the blue trajectory in Fig. 5, when it comes to oblique incidence from air to methane, whether the acoustic waves can go through the structure along PD depends on the incident angle and phase delay provided by the gradient structure. Consider that ${\theta }_{t2}=\mathrm{arcsin}\left[\frac{{n}_{\mathrm{M}}}{{n}_{\mathrm{A}}}\mathrm{sin}{\theta }_{cr}+\frac{\mathrm{d}\varphi \left(x\right)}{\mathrm{d}x}\frac{{\lambda }_{\mathrm{A}}}{2\pi {n}_{\mathrm{A}}}\right]$ from Eq. (1), where ${\theta }_{cr}$ and ${\theta }_{t2}$ are the critical incident angle in methane and transmitted angle in air respectively. When ${\theta }_{cr}$≥10° is satisfied, the output angle of acoustic waves is greater than 90°, which means the oblique incident acoustic waves with ${\theta }_{i2}$≥7° are blocked by the gradient structure.

The acoustic waves incident from left (shown by the red trajectory in Fig. 5) satisfy the expression: ${n}_{\mathrm{A}}\mathrm{sin}{\theta }_{i3}+\frac{\mathrm{d}\varphi \left(x\right)}{\mathrm{d}x}\frac{{\lambda }_{\mathrm{A}}}{2\pi }={n}_{\mathrm{M}}\mathrm{sin}{\theta }_{t3}$. Since $\frac{\mathrm{d}\varphi \left(x\right)}{\mathrm{d}x}\frac{{\lambda }_{\mathrm{A}}}{2\pi }$=0.91 and ${n}_{\mathrm{M}}\mathrm{sin}{\theta }_{t3}$<0.73. As a result, regardless of the incident angle ${\theta }_{i}$, the transmitted angle ${\theta }_{t3}$ has no solution, suggesting that the acoustic waves incident along ND cannot be transmitted.

**Figure 6:**
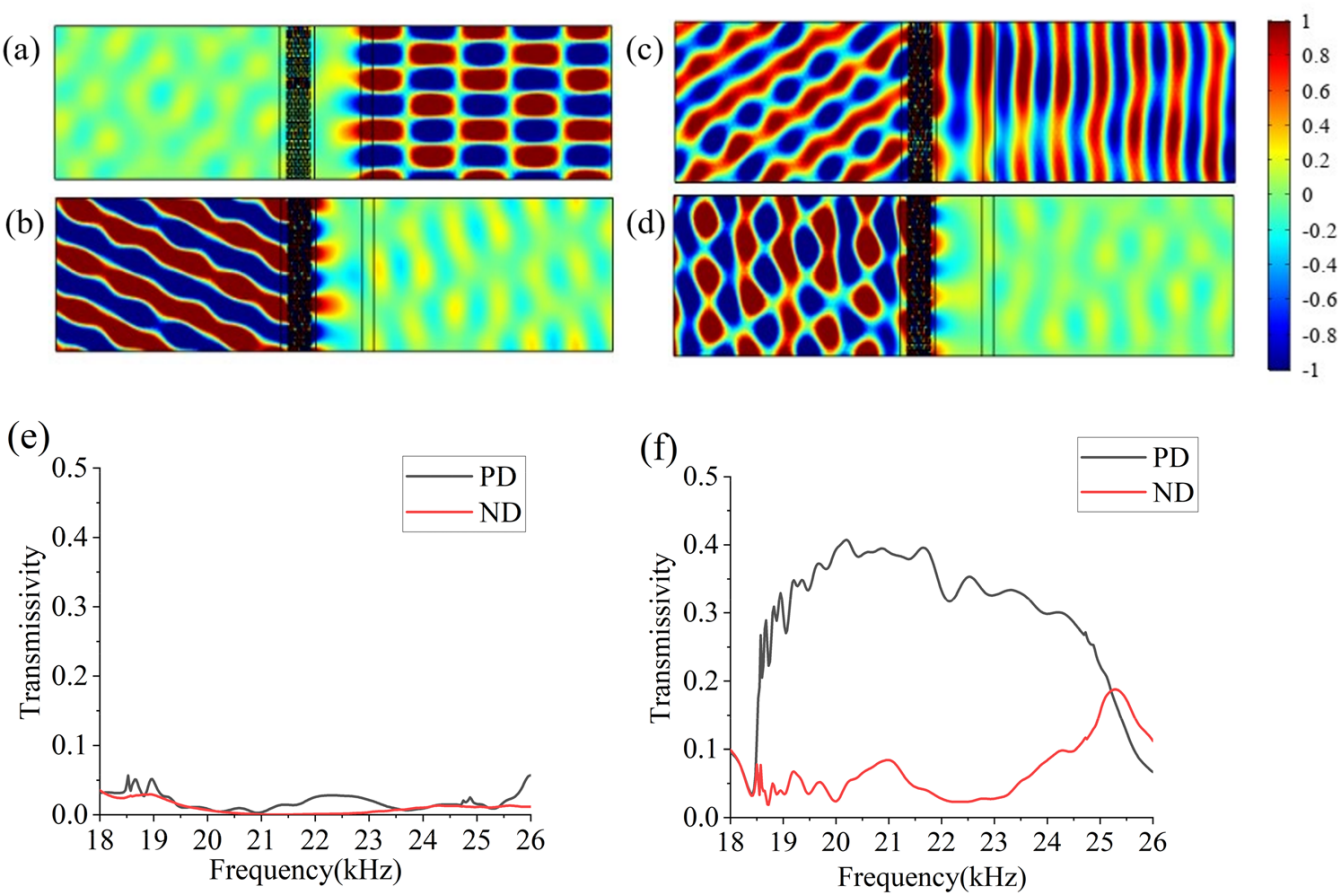
(**a–d**) Spatial distribution of the acoustic pressure generated by the oblique incident waves at 20 kHz: (**a,b**) incident angle of 60° in PD and ND; (**c,d**) incident angle of 3° in PD and ND. (**e,f**) Curves of transmissivity with frequency at 60° and 3°, the black and the red lines indicate the PD transmissivity and ND transmissivity respectively.

As shown in Fig. 6a,b, when the incident angle of acoustic waves at 20 kHz is above 7°, an acoustic insulation structure is formed. However, when the incident angle is lower than 7°, transmission situation acts as an asymmetric acoustic device, as shown in Fig. 6c,d. From Fig. 6e,f, it is obviously seen that the transmittance of acoustic waves with an incident angle of 60° from both sides is almost less than 0.05 over a wide frequency range; the PD transmissivity of acoustic waves with an incident angle of 3° is maintained above 0.3 and the ND transmissivity is below 0.1 mostly from 18.5 to 23.5 kHz.

To sum up, within the frequency range centered at 20 kHz, only oblique incident waves those who have the slight tilting can pass through the structure smoothly in PD but be blocked in ND, thus an acoustic asymmetric device is realized. On the other hand, when the incident angle is greater than 7°, the structure acts as a bidirectional acoustic barrier.

## Conclusion

In summary, we have designed a planar acoustic device composed of phase gradient structures for realizing angle-dependent asymmetric transmission. We theoretically deduce the condition for asymmetric transmission as $\frac{2\pi f}{{c}_{\mathrm{M}}}<\frac{\mathrm{d}\varphi \left(x\right)}{\mathrm{d}x}<\frac{2\pi f}{{c}_{\mathrm{A}}}$, which is verified by the simulation results showing the designed device produces asymmetric transmission for normal incidence waves within the predicted frequency ranging from 18.5 to 23.5 kHz. Based on this, we further investigate the dependence of the device’s performance on the incident angle. It turns out that when the incident angle is lower than 7°, the device only allows the acoustic waves coming from the right side to pass, giving rise to unidirectional transmission. However, acoustic waves with incident angle exceeding 7° will be blocked along both directions. Therefore, the designed device can be switched between a one-way device and a high-efficiency sound barrier by controlling the incident angle. We envision the realization of angle-dependent acoustic asymmetric transmission to enrich the physics of metamaterial-based sound manipulation and to find application in diverse fields such as noise control, medical diagnosis, etc.

## Method

Throughout the paper, the numerical simulations are conducted with commercial software COMSOL Multiphysics. The background medium I is air for which the mass density and sound speed are 1.21 kg/m^3^ and 343 m/s respectively. Medium II is methane with the mass density and sound speed being 0.78 kg/m^3^ and 467 m/s respectively. The material of the gradient phase structure in simulation is chosen to be polylactic acid (PLA) whose mass density and sound speed are 1250 kg/m^3^ and 2500 m/s, respectively. Plane wave radiation boundary conditions are imposed on the boundaries to eliminate the reflected waves by the outer boundaries and the periodic boundary conditions are employed on the upper and lower sides of the phase gradient profile in Figs. 3a–d and 6a–d.

## Data Availability

All data during this study are available within the paper. Additional data related to this paper are available from the corresponding authors upon reasonable request.
